# Ischemic Cardiomyopathy and Heart Failure After Acute Myocardial Infarction

**DOI:** 10.1007/s11886-022-01766-6

**Published:** 2022-08-16

**Authors:** Marco Giuseppe Del Buono, Francesco Moroni, Rocco Antonio Montone, Lorenzo Azzalini, Tommaso Sanna, Antonio Abbate

**Affiliations:** 1 Department of Cardiovascular Medicine, Fondazione Policlinico Universitario A. Gemelli IRCCS, Largo Agostino Gemelli, 1, 00168 Rome, Italy; 2 Department of Cardiovascular and Pulmonary Sciences, Catholic University of the Sacred Heart, Rome, Italy; 3 Berne Cardiovascular Research Center, University of Virginia, Charlottesville, VA USA; 4 Division of Cardiology, Department of Medicine, University of Washington, Seattle, WA USA

**Keywords:** Ischemic cardiomyopathy, Coronary artery disease, Myocardial infarction, Heart failure, Remodeling, HFrEF

## Abstract

**Purpose of Review:**

Ischemic cardiomyopathy refers to systolic left ventricular dysfunction in the setting of obstructive coronary artery disease and represents the most common cause of heart failure worldwide. It is often the combination of an irreversible loss of viable mass following an acute myocardial infarction (AMI) with a dysfunctional, but still viable, myocardium in the context of a chronically reduced myocardial blood flow and reduced coronary reserve. Medical treatments aiming at modulating neurohumoral response and restoring blood flow to the ischemic cardiomyocytes were shown to dramatically abate the occurrence of ventricular dysfunction and adverse remodeling in ischemic cardiomyopathy.

**Recent Findings:**

Novel therapeutic approaches, such as mechanical unloading and modulation of the inflammatory response, appear to be promising. Furthermore, the understanding of the mechanisms by which, despite optimal treatment, heart failure ensues after AMI, with or without adverse remodeling and systolic dysfunction, is a critical step in the search for novel ways to tackle heart failure risk beyond preservation of left ventricular volumes and systolic function.

**Summary:**

In this review article, we explore the principal pathophysiological mechanisms and pathways of heart failure in ischemic cardiomyopathy, therapeutic opportunities, and knowledge gaps in this area.

## Introduction

Ischemic cardiomyopathy refers to systolic left ventricular (LV) dysfunction in the setting of obstructive coronary artery disease (CAD), and represents the most common cause of heart failure (HF) worldwide [1]. Left ventricular dysfunction in patients with CAD is often the consequence of an irreversible loss of viable mass following an acute myocardial infarction (AMI), occasionally in combination with loss of contractility in ischemic, but still viable, myocardium (hibernating myocardium) [1, 2]. This has relevant therapeutic and prognostic implications since loss of contractility in hibernating myocardium is potentially reversible upon resolution of ischemia [3]. Irrespective of the underlying mechanism, patients with ischemic cardiomyopathy may have some degree of HF symptoms. The improvements in the acute treatment of myocardial infarction, revascularization strategies, and guideline-directed medical therapies led at reducing the incidence and degree of systolic dysfunction and adverse remodeling following AMI, yet the risk of HF remains substantial, suggesting that other mechanisms beyond LV systolic dysfunction and remodeling may also be implicated in HF symptoms following AMI [4–6, 7•, 8•, 9, 10].

In this review article, we explore the principal pathophysiological mechanisms and pathways of HF in ischemic cardiomyopathy, therapeutic opportunities, and knowledge gaps in this area.

## Determinants of Left Ventricular Dysfunction in Ischemic Cardiomyopathy

### Infarct Size

The infarct size following AMI is the most important predictor of LV dysfunction and remodeling after AMI [11, 12]. Larger infarct size due to late presentation or late reperfusion, no or minimal collateral flow, or anterior location is associated with greater LV dysfunction, adverse cardiac remodeling, and HF over time [13]. In a patient-level meta-analysis of 10 randomized trials including patients undergoing primary percutaneous coronary intervention (PCI), infarct size was strongly associated with subsequent mortality and hospitalization for HF [14].

### Myocardial Stunning

The degree of LV dysfunction during the acute phase of AMI is not only the result of the irreversibly myocardial loss but may be greater due to the contribution of myocardial “stunning,” regions of depressed cardiac function following the reperfusion of an occluded artery that gradually recover over the course of a days following a successful reperfusion [15]. The underlying mechanisms involve oxygen radical damage following reperfusion and altered calcium flux with calcium overload that then desensitizes the myofilaments [16]. This phenomenon, initially described following reperfusion using thrombolytic therapy, has also been shown to play a role in the current era of primary PCI, and contributes to the overall LV dysfunction [15].

### Coronary Microvascular Injury

Coronary microvascular injury following the recanalization of an occluded epicardial coronary artery is another important determinant of the final infarct size, and a predictor of future adverse remodeling and outcomes [17, 18]. This phenomenon has now been better characterized using CMR as a typical pattern with contrast-enhanced infarct area and contrast-void infarct core, known as “microvascular obstruction” (MVO) [17]. MVO is associated with further cardiomyocyte injury and inflammation through the development of a severe microvascular damage, which can cause the occurrence of intramyocardial hemorrhage with red blood cell extravasation and residual iron deposits triggering intramyocardial inflammation [17]. The mechanisms involved in the pathogenesis of coronary microvascular injury include ischemia- and reperfusion-related injury, distal embolization, and individual susceptibility.

All these mechanisms contribute to a worse prognosis by causing a larger infarct size, myocardial dysfunction, and adverse remodeling [19].

### Changes in Hemodynamics and Neurohormonal Activation

After AMI, the loss of contractile myocytes and reduced stroke volume leads to LV dilatation. This phenomenon can be acutely beneficial by maintaining stroke volume through the Frank-Starling mechanism. However, the LV wall stress is proportional to the radius of the ventricular cavity and inversely correlated to LV wall thickness, as explained by Laplace law [20]. Hence, LV dilatation increases wall stress and therefore oxygen consumption, leading to subendocardial myocardial ischemia via impaired coronary perfusion pressure, which fosters impaired LV contractility, reduced cardiac output, and the activation of adverse neurohormonal pathways over time. As part of the adverse ventricular remodeling post-AMI, cardiac geometry changes from elliptic shape to a spherical one, which is mainly driven by hypertrophic myocyte elongation in the non-infarcted zone, resulting in increase in wall mass and LV enlargement [20]. Furthermore, remote myocardial dysfunction could be seen as secondary to morphological changes in the infarct region, leading to an increased systolic longitudinal wall stress and loss in global ventricular function [21]. A relative increase in LV end-diastolic volume (LVEDV) of at least 20% using echocardiography has been traditionally used to define LV adverse remodeling. Compared with patients without adverse remodeling, those experiencing adverse remodeling had a greater risk of the composite of cardiovascular death, AMI, HF, stroke, or resuscitated cardiac arrest. This global adverse remodeling response leads to increases in both LV end-diastolic and end-systolic volumes and reduced LV ejection fraction (LVEF) [22–24]. Myocardial structural and functional changes in patients with ischemic cardiomyopathy are summarized in Table 1.

**Table 1:** Myocardial structural and functional changes in patients with ischemic cardiomyopathy

**Infarcted segment**
Edema
Scar formation
Hypo-a-kinesis → dyskinesis
Wall thinning → aneurysm formation
**Border zone segment**
Increase wall stress → infarct expansion
Hypertrophy
**Non-infarcted segments**
Hyper-kinesis
Increased wall stress
Hypertrophy
**Global remodeling**
Dilatation
Eccentric hypertrophy
**Systolic function**
Regional → global dysfunction
**Diastolic function**
Impaired relaxation
Elevated filling pressures
**Dimensions and function of the atria**
Atrial enlargement
Atrial fibrillation
**Valvular function**
Functional mitral regurgitation
Functional tricuspid regurgitation
**Electrical function**
Conduction block (AV block, BB block)
Increased automaticity
Re-entry tachycardia
**Neuro-autonomic function**
Increased sympathetic tone
Decreased parasympathetic tone
**Systemic vascular function**
Increase systemic vascular resistance
Reduced venous capacitance
**Pulmonary vascular function**
Pulmonary venous congestion
Post-capillary arterial hypertension
Reactive pre-capillary arterial hypertension

Reduced cardiac output promotes the activation of renin–angiotensin–aldosterone system (RAAS). Angiotensin II alters gene expression contributing to myocyte growth, affects cardiac metabolism and energetics, and increases protein synthesis in both fibroblasts and myocytes, thus increasing extracellular matrix deposition and fibrosis [25].

### Inflammation

AMI is a trigger for the inflammatory response aimed at clearing necrotic debris, which is then followed by an anti-inflammatory reparative phase leading to myocardial scar formation. Perturbation of this balance may acutely worsen the infarct size and chronically contributes to adverse remodeling and post-AMI [26]. While some degree of response is necessary to recruit leukocytes and clear the tissue debris, inflammation becomes itself a mechanism of disease. A sustained and dysregulated inflammation promotes cardiomyocyte death (through apoptosis or pyroptosis), impairs contractile function of the surviving cardiomyocytes through the cardiodepressant effects of some cytokines (i.e., interleukin 1), and promotes the disruption of the interstitial tissue hampering the formation of a structured infarct scar [26]. Elevated levels of C-reactive protein (a marker of systemic inflammatory response) have been associated with worse prognosis in patients with AMI and HF [27–29]. The inflammatory response is proportional to the degree of myocardial injury, so that larger AMI are associated with a greater inflammatory response.

### Hibernating Myocardium

LV dysfunction in the setting of CAD may also occur in patients without history of AMI. Repeated episodes of stunning as well as chronic low flow may induce metabolic and cellular adaptation leading to development of dedifferentiation of cardiomyocytes (hibernating myocardium) to prevent myocardial necrosis. Hibernating myocardium is viable myocardium, and therefore retains the ability to recover function upon revascularization [3]. The most common cause for the development of this condition is that a region of the myocardium is supplied by a stenotic coronary artery in which enough blood supply is available to maintain viability but not enough to maintain normal contractility of the region [2].

### Other Factors

Besides scar and stunning/hibernation, LV dysfunction in patients with CAD may also be the result of other contributing, pre-existent, or superimposed factors, such as ventricular interdependency, electromechanical desynchrony (i.e., left bundle brunch block), prior scar, or other causes of cardiomyopathies (genetic, metabolic, or toxic) that may coexist and meaningfully contribute to LV remodeling, dysfunction, and HF syndrome.

## Therapeutic Options

Several treatment options are currently available, and supported by strong evidence, in order to prevent or treat HF in ischemic cardiomyopathy. Prompt reperfusion of the infarct-related artery remains the mainstay treatment for AMI. As cardiomyocyte death per unit of time appears to be curvilinear with time from arterial occlusion, achievement of arterial patency in the first few hours after AMI allows for myocardial salvage and reduced infarct size, and subsequently might reduce the incidence of heart failure and other adverse outcomes [14, 30]. Thrombolysis was the first reperfusion strategy for AMI. In addition to be effective in abating in-hospital mortality [31], thrombolytic therapy was shown to significantly reduce infarct size by approximately 30% compared to placebo, with the largest benefit observed among patients who received thrombolysis within 1 h from the onset of symptoms [32]. Primary PCI was subsequently shown to allow for higher rates of effective revascularization compared to thrombolysis, which, combined with lower incidence of bleeding and reinfarction, led to a further improvement in outcomes for AMI patients [33]. In addition, primary PCI was shown to further reduce infarct size [34]. In patients with hibernating myocardium, revascularization of the viable myocardium is associated with improvement in cardiac systolic function, reverse remodeling, patient functional status, and HF symptoms. A comprehensive review of contemporary viability imaging techniques and their applications in clinical practice goes beyond the scope of this review article and can be found elsewhere [2].

Subsequent management strategies focus on modulating and interrupting the mal-adaptive process that led to infarct expansion and replacement fibrosis deposition within the cardiac muscle. Contemporary state-of-the-art pharmacological treatment focuses on modulation of the renin–angiotensin–aldosterone and of beta-adrenergic receptors. Of note, angiotensin-converting enzyme (ACE) inhibitors and angiotensin receptor blockers (ARB) were shown to reduce the development of myocardial fibrosis and hypertrophy, as well as LV adverse remodeling, hence reducing the onset of HF [35]. Table 2 summarizes the current indications to neurohormonal blockade in patients with ST elevation AMI.

**Table 2:** Medical therapy to prevent or treat ischemic cardiomyopathy in patients after STEMI

**Medication**	**Indication**	**AHA 2013** [72]			**ESC 2017** [73]	
		**COR**	**LOE**		**COR**	**LOE**
Beta-adrenergic receptor blocker(s)	**Oral beta-blockers should be initiated in the first 24 h in patients with STEMI who do not have any contraindication**	I	B	**Oral BB is indicated in patients with heart failure and/or LVEF <_40% unless contraindicated**	I	A
Angiotensin-converting enzyme inhibitor(s)	**All patients with STEMI with anterior location, HF, or LVEF less than or equal to 0.40, unless contraindicated**	I	A	**Within the first 24 h of STEMI in patients with evidence of heart failure, LV systolic dysfunction, diabetes, or an anterior infarct,**	I	A
				**ACE inhibitors should be considered in all patients in the absence of contraindications**	IIa	A
Angiotensin II receptor blocker(s)	**Patients with STEMI who have indications for but are intolerant of ACE inhibitors**	I	B	**Alternative to ACE inhibitors in patients with heart failure and/or LV systolic dysfunction, particularly those who are intolerant of ACE inhibitors**	I	A
Mineralocorticoid receptor antagonist	**All patients with STEMI and no contraindications who are already receiving an ACE inhibitor and beta-blocker and who have an LVEF less than or equal to 0.40 and either symptomatic HF or diabetes mellitus**	I	B	**In patients with an LVEF < _40% and heart failure or diabetes, who are already receiving an ACE inhibitor and a beta-blocker, provided there is no renal failure or hyperkalemia**	I	B

*COR* class of recommendation, *LOE* level of evidence, *STEMI* ST elevation myocardial infarction, *BB* beta-adrenergic receptor blocker(s), *LVEF* left ventricular ejection fraction, *ACE* angiotensin-converting enzyme, *HF* heart failure

Sacubitril-valsartan was recently studied in patients with AMI and high-risk features (LVEF ≤ 40% and/or pulmonary congestion at admission; STEMI 76%). At 2-year follow-up, sacubitril-valsartan failed to reduce the incidence of the combined primary of cardiovascular death, first HF hospitalization, or development of outpatient HF, which occurred in 11.9% of the sacubitril-valsartan group vs 13.2% of the ramipril group (HR 0.90; 95% CI 0.78–1.04, *p* = 0.17), despite the trends toward lower number of events for the individual endpoints with sacubitril-valsartan [36, 37]. Mineralocorticoid receptor antagonists have also been associated with better outcomes in patients with ST elevation AMI and reduced EF when initiated early after AMI [38, 39]. Beta-blockers indirectly modulate the renin–angiotensin–aldosterone system and may also have a direct antifibrotic effect at a cellular level, and thus were shown to reduce adverse remodeling and HF development after AMI [40]. Newer drugs, such as sodium-glucose cotransporter 2 (SGLT2) inhibitors, exert beneficial effects across of a wide spectrum of cardiovascular diseases, and are indicated among patients with HF regardless of the cause [41]. Ivabradine, a potent anti-anginal drug targeting heart rate hence reducing myocardial energy expenditure, was shown to have selective prognostic benefits in HF [42].

Considering the potential for functional recovery of the hibernating myocardium after perfusion restoration, revascularization has always been considered an attractive option for ischemic cardiomyopathy [43]. In fact, initial non-randomized studies showed a significant mortality benefit of coronary artery bypass graft (CABG) versus medical therapy among individuals with ischemic cardiomyopathy and a reduced LVEF [44]. The subsequent Surgical Treatment for Ischemic Heart Failure (STICH) enrolled 2112 patients with CAD and an LVEF ≤ 35% to assess the role of surgery in improving ventricular function and prognosis [45]. Among these, 1212 subjects with a coronary anatomy amenable to CABG were randomized to optimal medical treatment (OMT) or OMT plus CABG [45]. At 5-year follow-up, CABG resulted in significant reduction of death and hospitalization for cardiovascular causes (hazard ratio with CABG, 0.74; 95% CI, 0.64 to 0.85; *P* < 0.001) [45]. Long-term follow-up data showed a sustained benefit of CABG over OMT, resulting in a number needed to treat of 14 patients to prevent one death [46]. A significant interaction between CAD extent and outcomes was detected, with more extensive coronary artery involvement associated with greater benefit from surgery [46, 47]. In patients with ischemic cardiomyopathy, multivessel or left main CAD, and coronary anatomy not amenable to CABG, PCI should be considered even if there are not high quality data to support this approach. Notably, sub-analyses of randomized controlled trial showed that achievement of complete revascularization was associated to improved prognosis in ischemic cardiomyopathy [47, 48]. Based on this data, both American and European guidelines support revascularization when ischemia is believed to be a contributor to HFrEF [49, 50]. Whether the prognostic advantage is mediated by an improvement in myocardial contractility, electrical stabilization with the prevention of malignant ventricular arrhythmias, a reduction in ischemic events and cardiac death, or a combination of these factors is not currently known. In addition, extensive revascularization is associated with an improvement of LVEF [51]. A recently-published, real-world retrospective study including 10,071 patients from the Veteran Administrations showed that an increase in LVEF of more than 5% after revascularization was associated with significantly lower rates of mortality and HF hospitalization [52]. Similarly, a sub-analysis of the STICH trial including 618 patients showed that an increase in LVEF ≥ 10% after 24 months from enrolment was associated with better overall prognosis [53]. Interestingly, the occurrence of LVEF improvement was not different between patients who did or did not undergo revascularization, and CABG remained significantly associated to better outcomes even after adjusting for LVEF changes [53].

In patients with ischemic cardiomyopathy, an implantable cardioverter-defibrillator (ICD) and/or cardiac resynchronization therapy (CRT) pacemaker may be indicated. The current indications to ICD and/or CRT are found in detail elsewhere. CRT is indicated in particular in patients symptomatic for HF, in sinus rhythm, with a reduced LVEF (≤ 35%) and a QRS duration ≥ 130 ms [50]. QRS morphology (i.e., left bundle branch block), QRS width, sex (i.e., female), and low myocardial scar burden all predict a favorable response to CRT in terms of reverse remodeling and improved morbidity and mortality. Finally, for patients with severe chronic secondary (functional) mitral regurgitation, LVEF ≤ 50%, and New York Heart Association (NYHA) functional class II–IVa (ambulatory) HF despite optimum evidence-based management (pharmacologic therapy plus CRT, as indicated), transcatheter edge-to-edge repair can be considered if anatomically amenable [50].

### New Approaches to Ischemic Cardiomyopathy

Recent translational evidence has shown that unloading the LV through the use of a mechanical circulatory support device prior to revascularization during AMI has the potential to reduce the infarct size [54]. Proposed mechanisms for that include reduced LV work expenditure and oxygen consumption, activation of intracellular cardioprotective system, and increased collateral coronary circulation and myocardial perfusion [55]. The clinical implementation of a mechanical unloading strategy using intra-aortic balloon pump (IABP) was tested in the Counterpulsation and Infarct Size in Patients With Acute Anterior Myocardial Infarction (CRISP-AMI) trial [56]. The CRISP-AMI study recruited 337 patients with AMI, which were randomized 1:1 to receive IABP or not prior to PCI; the use of IABP was not associated to reduction in infarct size [56]. Pre-clinical data on transaortic unloading with microaxial pumps (i.e., Impella, Abiomed) appear to be more promising [57]. More recently, initial experience with LV unloading using Impella in humans has been published, showing excellent safety for this approach [58•]. Other percutaneous mechanical circulatory supports, such as transeptal centrifugal assist device (TandemHeart, Livanova) system, share similar hemodynamic effects with transaortic pump, including the unloading of the left ventricle by preload reduction, and may be have similar biological effect in terms of infarct size reduction, although data are currently lacking [59].

Similarly, the use of pressure-controlled intermittent coronary sinus occlusion (PiCSO), a mechanical catheter-based device placed into the coronary sinus after initial primary PCI, has shown to be effective in small, initial experiences in humans [60]. The system consists of a balloon-tipped catheter and a driving console, placed in the coronary sinus and provoking intermittent coronary sinus occlusion, hence redistributing blood flow to the border zone of deprived myocardium, enhancing the washout of deleterious agents, and increasing expression of vascular endothelial growth factor in the myocardium.

After revascularization, modulation of the inflammatory response appears to be an interesting target to improve infarct healing processes. Interleukin-1β (IL-1β) is a master cytokine in the cascade, released following activation of the inflammasome, which is able to induce local and systemic inflammation [61]. Notably, the inflammasome is activated in the setting of AMI, and the local activation of IL-1β was shown to foster ischemia reperfusion injury, favor infarction expansion, and overall increase the incidence of HF [61]. In the Virginia Commonwealth University Anakinra Remodeling Trial 3 (VCUART3), the administration of anakinra, recombinant IL-1 receptor antagonist, in the first 2 weeks after AMI significantly improved the incidence of HF or death [7•]. In particular, 26% of patients in placebo group either died or had new onset of HF, compared with 9% in the anakinra group, and 11% were hospitalized for HF as compared to 0% in the anakinra group [7•]. The Canakinumab Anti-inflammatory Thrombosis Outcome Study (CANTOS) trial randomized 10,061 patients with prior AMI and residual inflammation; canakinumab, a humanized IL-1β antibody or placebo given once every 3 months, subcutaneously showed to significantly reduce HF hospitalizations and HF-related mortality [62••]. Finally, a recent small trial Assessing the Effect of Anti-IL-6 Treatment in Myocardial Infarction (ASSAIL-MI) (*n* = 199) showed that tocilizumab, an inhibitor of the inflammatory cytokine interleukin-6 (IL-6) pathway, which is a downstream effector of IL-1β, increased the extent of myocardial recovery after reperfusion when compared with matching placebo [63••].

## New Paradigm of HF Without LV Dysfunction and Remodeling After AMI

**Fig. 1:**
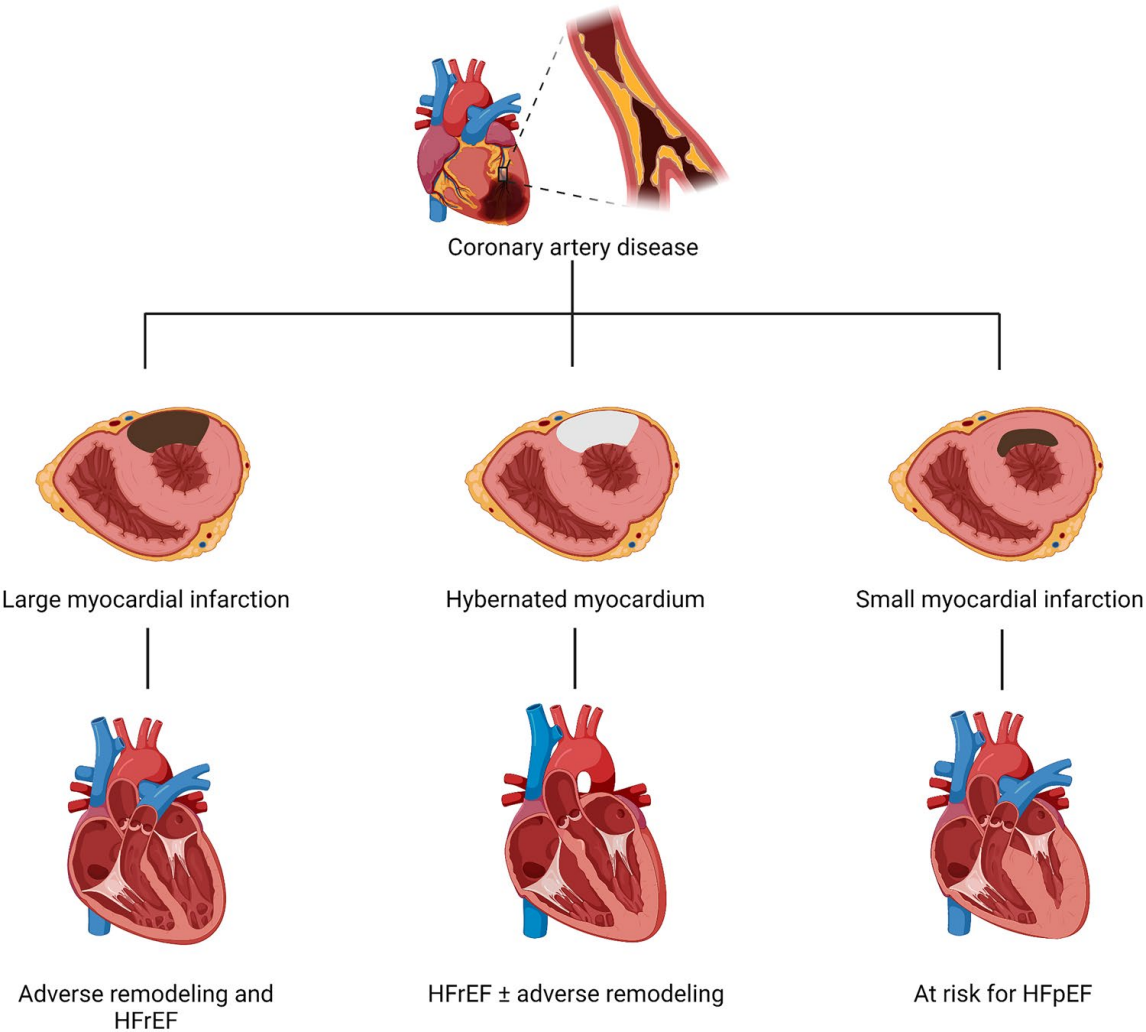
Heart failure and ischemic cardiomyopathy. Left ventricular dysfunction in patients with coronary artery disease (i.e., ischemic cardiomyopathy) is often the consequence of an irreversible loss of viable myocardium following a large AMI occasionally in combination with loss of contractility in ischemic, but still viable, myocardium (hibernating myocardium). However, even smaller infarcts, not necessarily associated with cardiac dilatation and dysfunction at rest, may put that patient at risk of developing HF with preserved EF as result of the combination of the post-infarction inflammatory, hemodynamic and neurohormonal response, and predisposing individual risk factors (i.e., obesity, older age, hypertension, chronic kidney disease, diabetes)

The advances in technology, reperfusion strategies, and the implementation of cardioprotective medications have led to a significant mitigation of LV systolic dysfunction and adverse cardiac remodeling after AMI. Strategies aimed at preventing LV dysfunction and remodeling may in fact also prevent the incidence of HF. HF after AMI is, however, described also in the absence of systolic dysfunction and adverse remodeling (Fig. 1) [64]. In a study including 374 patients undergoing CMR during hospitalization for STEMI and after 6 months, there was no significant change in LVEDV and LVEF at follow-up, but up to 7.5% of patients experienced a HF hospitalization at long-term follow-up [8•]. In a trial of colchicine versus placebo in STEMI, HF occurred in 13% of placebo patients despite minimal changes in relative remodeling at follow-up [9]. In the trial of cyclosporine versus placebo, 10% of STEMI patients experienced a HF hospitalization at 1 year, independent of changes in cardiac dimensions and systolic function (LVEDV and LVEF) [4]. In the aforementioned VCUART3 trial of anakinra versus placebo in patients with STEMI, the combined endpoint of new-onset HF and HF hospitalization or death occurred in 26% of patients in the placebo arm despite lack of changes in LVEDV and LVEF [7•]. In the pooled data analysis of three VCUART clinical including 139 patients with STEMI, all-cause death or new-onset HF occurred in 29.1% of placebo group at 1-year follow-up. Of these, only half of the patients had a reduced LVEF at follow-up (LVEF < 40%) [65].

Pathophysiologic mechanisms leading to HF in patients with CAD may be in fact multifactorial and only partially explained by measures of LV systolic dysfunction at rest or changes in LV volumes. The acute myocardial damage is only one of the factors implicated in the determination of cardiac contractility, LV filling pressure, and cardiac output reserve. Post-infarction inflammatory, hemodynamic and neurohormonal response, chamber compliance, myocardial stiffness, cardiac pre-existing individual risk factors, and subclinical comorbidities all contribute at vary degree to determine cardiac function and decline in cardiorespiratory fitness [66, 67]. Resting systolic function may be preserved but patients may still experience symptoms during exercise due to the loss of cardiac systolic and diastolic reserve, as it happens in HF with preserved LVEF [68–70]. Myocardial stiffness due to persistent chronic ischemia or previous subclinical functional structural changes may limit diastolic reserve and contribute to the reduced cardiorespiratory fitness [71].

## Conclusions

Ischemic cardiomyopathy is the most common cause of HF in the general population. Treatments aiming at modulating the neurohumoral response and, in selected cases, restoring blood flow to the ischemic cardiomyocytes were shown to dramatically abate the occurrence of HFrEF in ischemic cardiomyopathy. Novel therapeutic approaches, such as mechanical unloading and modulation of the inflammatory response, appear to be promising. Furthermore, the understanding of the mechanisms by which, despite optimal treatment, HF ensues after AMI, with or without adverse remodeling and systolic dysfunction, is a critical step in the search for novel ways to measure HF risk beyond preservation of LV volumes and LVEF, and for novel interventions to reduce the incidence of HF after AMI with subsequent consequences on morbidity and mortality. The scarcity of data exploring cardiac reserve and cardiorespiratory fitness following an AMI paves the way for more systematic investigations to provide novel pathophysiological insights and a better understanding of the problem.
